# Multifunctional Role of Chitosan Edible Coatings on Antioxidant Systems in Fruit Crops: A Review

**DOI:** 10.3390/ijms22052633

**Published:** 2021-03-05

**Authors:** Giuseppina Adiletta, Marisa Di Matteo, Milena Petriccione

**Affiliations:** 1 Department of Industrial Engineering, University of Salerno, Via Giovanni Paolo II, 84084 Fisciano, Italy; gadiletta@unisa.it (G.A.); mdimatteo@unisa.it (M.D.M.); 2 CREA-Centre for Olive, Fruit and Citrus Crops, Via Torrino 3, 81100 Caserta, Italy

**Keywords:** fruit crops, chitosan, postharvest, oxidative stress, enzymes, antioxidant

## Abstract

Chitosan-based edible coatings represent an eco-friendly and biologically safe preservative tool to reduce qualitative decay of fresh and ready-to-eat fruits during post-harvest life due to their lack of toxicity, biodegradability, film-forming properties, and antimicrobial actions. Chitosan-based coatings modulate or control oxidative stress maintaining in different manner the appropriate balance of reactive oxygen species (ROS) in fruit cells, by the interplay of pathways and enzymes involved in ROS production and the scavenging mechanisms which essentially constitute the basic ROS cycle. This review is carried out with the aim to provide comprehensive and updated over-view of the state of the art related to the effects of chitosan-based edible coatings on anti-oxidant systems, enzymatic and non-enzymatic, evaluating the induced oxidative damages during storage in whole and ready-to-eat fruits. All these aspects are broadly reviewed in this review, with particular emphasis on the literature published during the last five years.

## 1. Introduction

Fruits and vegetables, being an excellent source of nutrients, play an important role in human nutrition and high fruit and vegetable intakes in diet are also associated with a decreased incidence of a variety of chronic diseases [1]. Fruits and vegetables are very perishable commodities and their losses and wastes occur at various stages of food supply chain [2].

A challenge to the food industries is to develop and implement several innovative methods and technologies in order to control and reduce fruits and vegetables postharvest losses [3,4]. Edible coatings (ECs) represent an eco-friendly, low-cost, promising and effective method that create a thin layers of edible substances on fruit surfaces, exhibiting film-forming properties to prevent deterioration during postharvest life of whole and fresh-cut fruits and vegetables [5,6]. ECs should be realized by biodegradable and biocompatible materials recognized as “generally regarded as safe” (GRAS) or as food additives by the Food and Drug Administration and the European Union, respectively [7]. ECs create a physical protective barrier between the fruit and the environment modifying the permeability to gas exchange, water vapor and the atmosphere around the commodities [8]. Consequently, in coated fruits and vegetables, respiration and transpiration rates and physiological ripening process are slow down, while the qualitative and sensorial traits are enhanced [3].

Various types of biopolymer matrix, all biodegradable and non-toxic, such as polysaccharides, lipids and proteins are used alone or in blends to develop ECs. The coating materials are applied by spraying or dipping method on the surface of fruits and vegetables to obtain a protective mono- or bi-layer [9].

Polysaccharides are renewable biopolymers widely used in ECs applications for their solubility in water, high available and low allergen content. Furthermore, their mechanical and structural properties are due to a hydrophilic hydrogen-bond network with ordered and compact structure [10]. Pullulan, chitosan (CS), carrageenan, starch, alginate, cellulose, pectin, gellan gum, and xanthan gum are the main polysaccharide-based materials used for ECs [9].

CS based coatings are considered the best edible and biologically safe preservative coatings for different fruits and vegetables, owing to their lack of toxicity, biodegradability, and antimicrobial action [11].

This review is carried out with the aim to provide comprehensive and updated overview of the state of the art related to the effects of CS-based ECs on antioxidant systems, enzymatic and non-enzymatic, evaluating the induced oxidative damages during storage in fresh and ready-to-eat fruits. All these aspects are broadly reviewed in this review, with particular emphasis on the literature published during the last five years (Table S1).

## 2. Chitosan: Structure, Functional Properties, and Methods of Application

CS is a deacetylated derivate of chitin whose is the second-most abundant renewable biopolymer in nature. CS is a cationic linear polysaccharide composed of β-(1–4)-linked D-glucosamine (deacetylated unit) and N-acetyl-D-glucosamine (acetylated unit), derived from the structural components of arthropod exoskeletons or the cell walls of fungi and yeast [11]. This polymer is soluble in acidic to neutral solutions and shows positive charges due the protonation of its amino groups, whose pKa value is equal to 6.5 [12].

Percentage of deacetylation, different acid compounds, and molecular weight influence the solubility, physical, or antimicrobial properties and film thickness of CS [13].

CS has antimicrobial activity against different microorganisms such as Gram-positive and Gram-negative bacteria, fungi, and yeast. Antimicrobial efficacy has explained by several mechanisms of action although the most cited and suggested mechanism is due electrostatic interaction among this compound and microorganisms [14]. At low pH values a high electrostatic interaction between protonated amino (-NH_2_) groups of CS and the anionic carboxyl and phosphate groups of bacterial outer surfaces is observed. These interactions not only alter the permeability of the bacterial cell membrane, impeding gas exchange between the interior of a cell and the exterior environment but also cause cellular dysfunction through the rupture of the membrane and release of intracellular components [15]. The main factor responsible of CS antimicrobial activity is the presence, the density and the location of cationic charges in the polymer backbone [14]. Furthermore, antimicrobial properties are also correlated to CS molecular weight (MW) and degree of acetylation (DA) as suggested by Sharma et al. [16] that demonstrated as CS with lower MW and DA controls more effectively the microbial growth rate [16]. CS with an average MW above 60 kDa inhibits the growth of several species of Gram-positive and Gram-negative bacteria with dissimilar efficiency due to the different composition of the cell wall [14]. Due to its low toxicity and antimicrobial properties, CS has been approved by European Union for plant protection (Reg. EU 2014/563) [15].

CS-based coatings can be applied on surface of fruits and vegetables by several deposition methods such as dipping and spraying. Nature and surface of commodities influence the diffusion of the CS coating solution during adhesion procedure. Furthermore, a high affinity of coating solution for the product reduce the contact time during wetting stage [17].

Dipping is the method widely used to applied CS-based coatings submerging the fruits and vegetables in an acidic CS solution which may contain the natural preservative (s) and plasticizer(s) [12]. Commodities are immersed in the coating solution to ensure an effective interaction between substrate and the formulation of edible coating. Afterwards, by a deposition process a thin layer of the precursor emulsion is developed on the surface of product and the excess liquid is drained and solvent is evaporated at room temperature or by using heating and drying procedures, forming the thin coating [9,18]. This method is simple and convenient, it does not require sophisticated equipment and it is widely used to apply edible coatings to fresh produce. Furthermore, the dipping method ensures good uniformity across a rough and complex shape on the surface of food products [12].

Spraying methods allow to form a uniform thickness coating increasing the liquid surface through forming droplets by a set of nozzles. Furthermore, this technology is the most common method used in the application for coating on food products avoiding the contaminations of commodities and offering the possibility for multilayer applications with alternating solutions. Food industries use three types of spraying techniques such as air spray atomization, air assisted airless atomization, and pressure atomization [9].

CS coatings can be applied on surface of fruit and vegetables as mono and multilayer applications [13]. In multilayer edible coating is used layer-by-layer (LbL) electrostatic deposition technique, where a thin coating is formed by multiple dipping in two solutions with oppositely charged polyelectrolytes separated by drying step to remove the excess of coating solution in whole or fresh-cut fruits and vegetables [13]. The self-assembly of each coating is driven by the electrostatic force generated by the change in charge on the coating surface and play an important role in the overall performance of the multi-coat systems [19]. The electrical charge could be changed by pH, which significantly affects the amount of deposition of the polymers, as more biopolymer molecules are required to neutralize the previous layer [12].

**Figure 1:**
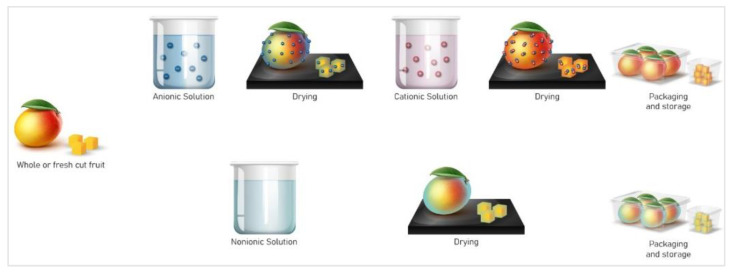
Principal steps involved in mono and bilayer chitosan (CS)-based edible coatings in whole and fresh-cut fruits.

CS-based multilayer coatings are valid tools to prolong the shelf life in whole and ready-to-eat fruits, and they take advantages of each single-layer coating with complete structure and stable performance [16,20] (Figure 1). In particular, multilayer coatings have become of interest in fresh-cut samples that show hydrophilic properties on the cut surfaces and not allowing a good coating adhesion with a single dipping [9]. A multilayer CS-based coating, enriched with essential oils, has been create combining microcapsule technology, LbL electrodeposition techniques, and coating technology [19].

CS can be used to realize nanoparticles with excellent physicochemical characteristics such as high surface area and charge density with antimicrobial and antioxidant properties [21]. Nanoparticles are used as a method for the integration and storage of active substances in different fields and consist of macromolecular and/or molecular structures in which active substances are dissolved, stored, encapsulated, or even adsorbed, or maintained on the external interface. There are several methods to produce CS nanoparticles as well as ionic gelation, emulsification and crosslinking, complexation with polyelectrolytes, self-assembly, and drying processes [22].

## 3. Reactive Oxygen Species and Antioxidant Systems

Reactive oxygen species (ROS), such as superoxide anion (O_2_^−^), hydroxyl radical (OH), hydrogen peroxide (H_2_O_2_), and singlet oxygen (^1^O_2_), are generated from cellular processes such as photosynthesis and respiration in several cellular organelles and they are formed as by-products of normal aerobic metabolism or as response to abiotic and biotic stresses [23]. ROS have always been known as harmful compounds, involved in lipid peroxidation and consequently responsible to membrane damages as well as in oxidation of other biological molecules such as proteins, DNA and carbohydrates (Wang et al. 2019). In the last years, several studies have demonstrated that ROS have a signaling role in the modulation of a variety of crucial pathways in fruit physiological processes such as development and ripening [23,24]. ROS double role leads to define these compounds as “a double-edged sword of life” [25].

ROS network is maintained at steady-state level by strong antioxidant system that regulate the scavenging levels based on ROS production in the fruit [26]. The antioxidant system is mainly divided into an enzymatic antioxidant system and a non-enzymatic antioxidant system [27].

Endogenous radical-scavenging antioxidants in fruits are vitamin E and A, ascorbic acid (vitamin C; AA), flavonoids, carotenoids, glutathione (GSH), polyphenols, allyl sulfides, curcumin, melatonin, and polyamines and they are involved in the detoxification of ROS as well as O_2_^−^ and OH [28,29,30]. These antioxidants can be water-soluble and predominantly located in the cytosol and cytoplasm or liposoluble and are present in cell membranes [31].

Enzymatic antioxidant system includes superoxide dismutase (SOD; EC 1.15.1.1), catalase (CAT; EC 1.11.1.6), ascorbate peroxidase (APX; EC 1.11.1.11), guaiacol peroxidase (GPX; EC. 1.11.1.7) and represents the frontline defense antioxidants. SOD is the first ROS scavenging enzyme which catalyzes the dismutation of O_2_ into H_2_O_2_ and molecular oxygen (O_2_) through a cyclic oxidation–reduction electron-transfer mechanism [32]. H_2_O_2_ is detoxified by CAT and APX, belonging two different classes of H_2_O_2_-scavenging enzymes with different affinities to the substrate. CAT dismutates H_2_O_2_ to oxygen and water but it has a low substrate affinity. APX is the first enzyme of ascorbate-glutathione cycle also known as Asada–Halliwell pathway, it has a high substrate affinity and is involved in regulating H_2_O_2_ as a signaling molecule while CAT is directly related to H_2_O_2_ detoxification [33]. APX reduces H_2_O_2_ to water by utilizing ascorbate as an electron donor which is also converted into monodehydroascorbate (MDHA) [34].

**Figure 2:**
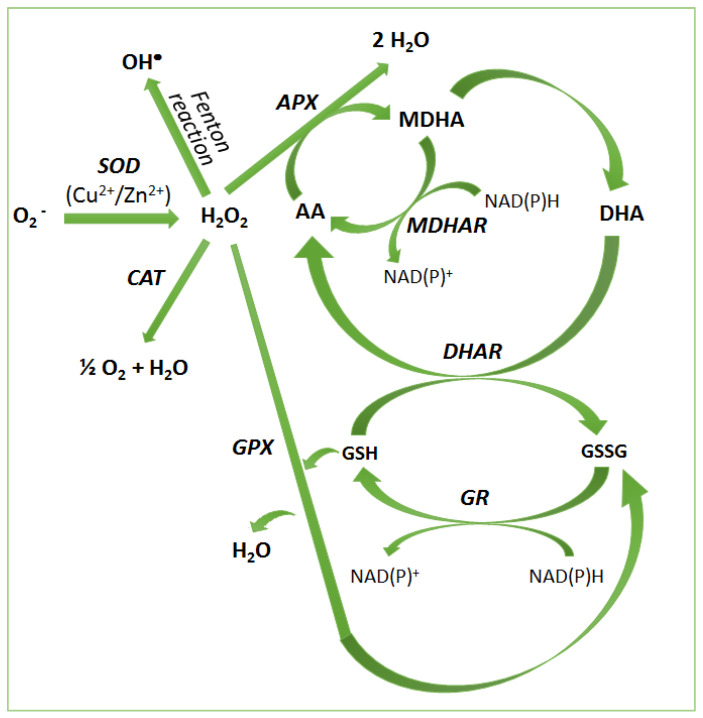
Enzymatic antioxidant systems to control redox homeostasis. Superoxide dismutase (SOD), catalase (CAT), ascorbate peroxidase (APX), glutathione peroxidases (GPX), monodehydroascorbate reductase (MDHAR), dehydroascorbate reductase (DHAR), glutathione reductase (GR), ascorbate (AA), monodehydroascorbate (MDHA), dehydroascorbate (DHA), oxidized glutathione (GSH), reduced glutathione (GSSG).

MDHA is spontaneously dismutated into dehydroascorbate (DHA) or converted into AA by the activity of monodehydroascorbate reductase (MDHAR, EC 1.6.5.4) using NADPH as the electron donor. DHA is reduced to AA again by dehydroascorbate reductase (DHAR, EC 1.8.5.1) using GSH, which is oxidized into glutathione disulfide (GSSG). This latter is reduced into GSH by the activity of glutathione reductase (GR, EC 1.8.1.7) using NADPH as the electron donor (Figure 2) [35].

All enzymes, associated with the AA-GSH cycle, are important to maintain AA and GSH redox state and to confer stress tolerance in plants [36].

### 3.1. Effect of Chitosan on ROS Content and Oxidative Membrane Damage

CS-based coatings reduce ROS content in fruit cells during postharvest life. In fact, in CS/nano-silica treated loquat a reduction around ~35% and ~69% in the O_2_^•−^ and H_2_O_2_ content has been observed [37], while in mango and nectarine coated with high MW CS a significant reduction (*p* ≤ 0.05) in H_2_O_2_ content has been registered [8,38]. Similarly, H_2_O_2_, O_2_^−^ and OH content, in apple slices treated with S-nitrosoglutathione-CS nanoparticles (GSNO-CS NPs) represented 50.20%, 48.67%, and 27.63% of that in the control after 4 days of cold storage, respectively [32,39] showed that GSNO-CS NPs coated sweet cherry contained a significantly lower level of ROS content at the end of storage. Similarly, in CS-based coated majiayou pummelo the H_2_O_2_ and O_2_^−^ content were lower than control of 25.4% and 19.2%, respectively [40].

CS in combination with organic acid such as citric, ascorbic, malic, and ossalic acid significantly reduced H_2_O_2_ and O_2_^•−^ production in coated cherimoya and pomegranate fruits [41,42].

Furthermore, Jiao et al. [43] have also been detected a high ROS intracellular level by histo-chemical staining with 2,7-dichlorodihydrofluorescein diacetate (DCFH-DA) fluorescent probe, mainly located in cell membrane and nucleus in peach control samples compared to CS grafted with chlorogenic acid coated fruit [43].

ROS accumulation can lead to widespread oxidative stress with oxidation of membrane lipids due to lipoxygenase (LOX) activity, leading to a rapid reduction of fruit quality and marketability. LOX activity catalyzes deoxygenation of polyunsaturated fatty acids to produce toxic fatty hydroperoxy acids. Malondialdehyde (MDA) and membrane permeability are two direct biomarkers used to evaluate the lipid peroxidation and the structural integrity of the membrane, respectively [34].

MDA is the final product of lipid peroxidation and its content is a direct indicator of cell membrane injury and it can be used to assess the index of cell oxidative damage. MDA content increases during fruits postharvest life due to physiological ripening process that improves the respiration rates leading to ionic leakage and accumulation of ROS [44]. CS-based coatings alleviate the membrane lipid peroxidation reducing ROS accumulation and LOX activity, as indicated by the lower MDA content, keeping quality of coated fruits during storage (Table S1).

Several studies demonstrated that CS-based coating reduced the MDA increase in strawberry [45,46,47,48,49], mango [50,51], loquat [37,52], grape [38,53], lemon [54], nectarine [55], pomegranate [42], plum [56], majiayou pummelo [40], cherimoya [41], sweet cherry [57], and apple [46] during storage. These findings suggest a low degree of lipid peroxidation in CS coated fruits.

In different studies have also been demonstrated that CS-coated fruits displayed significantly lower LOX activity than uncoated ones during storage, suggesting that CS coatings contributed to the maintenance of membrane integrity preserving cell compartmentalization [13]. In sweet cherry cultivars an increase in LOX activity has been registered during cold storage and shelf-life with lower values in CS coated fruit, “Della Recca” showed values slightly lower than “Ferrovia” and “Lapins” implying greater preservation of membrane integrity [57]. Interestingly, CS-nanosilica coated loquat fruit showed a relatively stable condition of LOX activity throughout cold storage while control fruit exhibited a sharp increase of this enzyme [37]. It has also been reported that shelf life at ambient air (27 °C) in guava fruit induced a gradually increase of LOX activity up to the ninth day and a slowly decrease until up to end of the experiment with lower values in CS-polyvinilpirrolidine (PVP)-salicylic acid (SA) (2 mM) coated fruit [58].

Furthermore, in strawberry and loquat fruits, CS-coating slowed down the increase of LOX activity during cold storage in all analyzed cultivars [45,52]. Likewise, a lower LOX activity has been registered in CS-coated grape during postharvest partial dehydration of “Sagrantino” fruit [53].

CS-based coating treatments in avocado fruit also induced an elicitation effect by showing a significant down-regulation of LOX genes in artificially fruit inoculated with Colletotrichum gloeosporioides after 14 days cold storage and 5 days of shelf life, or naturally infected fruit after 28 days of cold storage and 5 days of shelf life [59].

### 3.2. Effect of Chitosan on Antioxidant Systems

**Figure 3:**
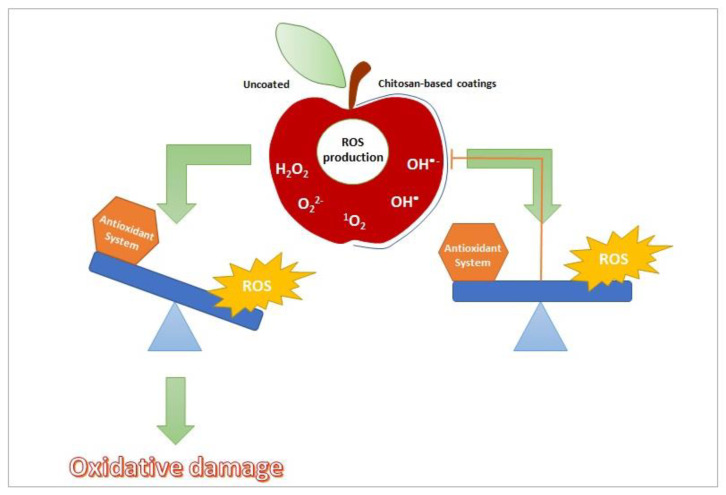
CS-based coating counteracts oxidative stress reducing ROS over-production through enhancing of antioxidative defense system.

CS-based coatings can counteract oxidative stress reducing ROS over-production through enhancing of antioxidative defense system including enzymatic and non-enzymatic components that work synergistically to improve cellular defense in fruits (Figure 3) (Table S1).

#### 3.2.1. Non-Enzymatic Antioxidant System

Fruits are an important and rich source of antioxidant compounds involved in defense systems that maintain a steady-state ROS level [60]. Non enzymatic antioxidants are small organic molecules that suppress the initiation and/or break the propagation of free radical chain reactions [31].

CS-based coatings act as a barrier film on fruit surface that preserve the non-enzymatic antioxidant content during storage [13]. Previous studies have demonstrated that CS-based coatings modify the atmosphere surrounding the fruit acting as semipermeable barriers that control gas exchange (C_2_H_4_, CO_2_, and O_2_) [61,62]. A low oxygen permeability in CS-coated fruits leads to the inhibition of the enzymes’ activity involved in oxidative reactions of bioactive compounds [63,64]. Several studies have demonstrated that a low oxygen permeability were registered in high MW CS coating in peach [55] and mango [8] fruits with significant changes in the respiratory pathway metabolism and reduction of ripening process by the suppression of ethylene production.

AA is considered an important antioxidant that reduces ROS and is converted to its oxidized form, DHA [19], but this compound is also a cofactor in many enzymes [65]. AA levels change throughout fruit ripening and storage in fruit crops-dependent manner due to different regulation of its metabolism [45,65]. CS-based coatings regulate the passage of oxygen from the external environment into the fruit, inhibiting the activity of ascorbate oxidase, responsible to the AA oxidation [38]. CS was found to be effective in restricting the losses of AA when applied as a coating on fruits such as plum [56], litchi [66], sweet cherry [11], longan [67], mandarin [68], majiayou pummelo [40], red kiwifruit [69], and pomegranate [64,70].

Several studies have been demonstrated that CS-based coatings improve the increase of AA content in loquat [71], sweet orange [72], and strawberry [45] fruits throughout cold storage. In guava fruit, CS-based coating with 3% of CS delayed the ascorbic acid biosynthesis during ripening fruit compared with the control [73]. The same results have been obtained in pomegranate arils coated with 0.5% and 1% of CS after 15 days of cold storage [74].

Furthermore, the application of CS coating enriched with lactoperoxidase, aloe vera gel, savory and/or tarragon essential oils, phenolic compounds, and organic acid on mango, litchi, fig, cherimoya, kumquat, and apple fruits reduced the losses of AA during storage, respectively [40,75,76,77,78,79,80]. Benitez et al. [81] also demonstrated that the effectiveness of the CS coatings in kiwifruit slices on oxidative degradation of AA depends on the organic acid used in the formulation of coating.

CS-based coatings enriched with olive (olive leaves and pomace), apple (peel) or pomegranate (peel) extracts, in strawberry and guava fruits maintain higher levels of AA than the uncoated samples, decreasing during storage [47,82,83]. Furthermore, montmorillonite (MMT) has been incorporated as nano-fillers into CS coatings, with different percentage, to investigate their effects on storage of tangerines and table grapes [84,85]. CS-MMT (2% *w*/*w*) coating on tangerine fruits delayed the postharvest ripening process and increased the AA content at the end of storage compared to other samples [85], while CS-MMT (0.1% *w*/*v*) coating significantly (*p* < 0.05) inhibited the decrease of AA in table grape after 20 days at 4 °C [84].

Layer-by-layer ECs obtained by a combination of CS and mucilages or CS and carboxymethyl cellulose gave the best performance, slowing down the AA losses in fresh-cut pineapple and lemon, respectively [24,86]. Zhang et al. [20] tested CS-based multilayer coatings, comprising pullulan/CS in order to preserve the postharvest quality of papaya. This fruit with four-layers of coating exhibited the highest AA content at the end of storage. Yin et al. [19] developed a multilayered edible coating based on CS (0.2 g/100 g) and Na-alginate (0.2 g/100 g) using as antimicrobial agent the encapsulated cinnamon essential oil to extend the shelf-life of mango during storage at 25 °C for 14 days. Cinnamon oil in the microcapsules of the CS coating possessed antioxidant properties, and its volatilization inhibited the oxidative breakdown of AA, maintaining a high AA content and a low DHA content compared to control in coated mango [19]. GSNO-CS NPs have been realized by S-nitrosoglutathione (GSNO) incorporation into CS coating and used to preserve sweet cherries and fresh-cut apple slices quality during cold storage [32,39]. GSNO-CS NPs may regulate both indirect and direct the AA-GSH cycle known as the indirect antioxidant system in apple slices and sweet cherry with an increase in AA and a decrease in DHA content compared to control [32,39]. Furthermore, CSNPs are loaded with Satureja hortensis essential oil and their efficacy as a coating layer have been tested on pomegranate arils quality. CSNPs-SH prevent the AA loss during storage by affecting arils water content [87].

Phenolic compounds, including flavonoids, are metabolites produced by secondary metabolism through phenylpropanoid pathway and possess strong free radical scavenging activity and chelating properties [60]. These compounds have numerous beneficial effects on human body protecting against different chronic diseases [88].

However, like AA, total phenols (TP) and flavonoid content (TF) decrease in fruit during postharvest storage [89]. CS-based coatings prevent the loss of phenolic compounds due to protective barrier effect [60]. Different DA and MW, such as storage temperatures, influence the TP content in CS coated fruits during storage [90]. In sweet cherry, CS based coating with high MW and low MW exhibited the highest TP content (554.88 mg/kg) at 4 °C and at 20 °C (518.77 mg/kg), respectively [90]. Likewise, nectarine treated with high MW CS showed better TP and TF content than low MW throughout the storage [54]. Drevinskas et al. [91] demonstrated that CS-based coatings with different MWs on kiwifruit had a different effectiveness in cultivar-dependent manner.

High polyphenols content has been registered in CS-coated plums compared to uncoated fruit throughout 35 days of cold storage [56]. It has also been reported that TP and TF content decreased in guava stored, but CS coated samples showed higher values compared to control [63]. CS coating improved the increase of TP content in pomegranate during 4 months of storage and delay the decrease at the end of cold storage [70]. In packaged “Herskawitz” and “Wonderful” pomegranate arils, TP decreased with the increase of storage, and a higher TP concentration was found in treated samples with higher CS concentration of 7.5 and 15 g/L [64].

In litchi fruit, TF had a rapid increase up to the second days of storage, followed by a rapid decline until the end of storage while TP content exhibited a rapid decrease from storage day 0 to day 2, followed by an increase from storage day 2 to day 4, and a final decrease. CS coatings maintained higher TF and TP content in litchi stored at 25 °C for 6 days [66]. Similarly, CS-based coating slowed down the decrease of TP content in red kiwifruit and longan during 26 days at 20 °C and 6 days at 25 °C, respectively [67,69] and TF content in strawberry at 4 °C for 15 days [47].

Other studies carried out by Petriccione et al. [11,45,71] demonstrated as CS-based coatings improved the bioactive compounds content, as well as TP and TF, of loquat, sweet cherry and strawberry fruits during cold storage for 21 days, 14 days and 9 days, respectively. Furthermore, CS-based coating maintained higher TP content during postharvest partial dehydration of “Sagrantino” grape [53].

The nutraceuticals content in several fresh fruits depends on CS coating alone and/or in combination with organic acid such as ascorbic, malic, citric, and ossalic acid that play important roles in physiological and metabolic respiratory pathways in fruits [41]. In pomegranate fruit, CS-based coating (2.0% *w*/*v*) in combination with malic (50 mM) and ossalic acid (5 mM), maintained higher levels of TP content with 12.74% and 14.65% differences compared to control, respectively [42]. Furthermore, in pomegranate arils stored TP content was excessively improved by organic acids and CS coatings up to 5 days [74]. Similarly, Adiletta et al. [75] revealed that fig fruit treated with CS-based coating combined with ascorbic acid (1% *w*/*v*) exhibited higher TP content stored at 4 °C for nine days. Likewise, Özdemir and Gökmen [92] reported that application of CS and ascorbic acid based coating showed higher content and slower decline of TP content compared with the control by the end of storage in fresh-cut apples.

CS coatings enriched with salicylic acid into the backbone chain inducing the formation of a CS-g-salicylic acid (CS-g-SA) conjugate, shown to have an effective role to preserve bioactive compounds in litchi [78], table grape [93] and pomegranate [94] fruits. Litchi treated with SA (1.0 mM) in combination with CS (2% *w*/*v*) retained about 75% of initial phenolics after 6 days of storage and allow to maintain the highest TF content at the end of storage [78]. Furthermore, CS-g-SA coating induced greater accumulation of TP in table grape and pomegranate and preserved them significantly better than the uncoated samples [93,94]. Slower reduction of TP and TF content in CS-g-SA and CS treated fruits than control might be attributed to antisenescence properties of SA and CS [78] or to higher phenylalanine ammonia-lyase (PAL) activity [94]. CS-SA-treated pistachio displayed the highest TP content at the end of 28 days of storage [95]. Likewise, CS/PVP-SA (2 mM) coating showed a high effectiveness to minimize the TP content losses during storage in “Banati” guava [58]. Jing et al. [89] also found that procyanidin-grafted CS maintained a higher TP content in fresh-cut pineapple than both CS coated and uncoated samples.

CS coating combined with UV-C irradiation was able to retain a higher level of TP content in longan fruit during 7 days at 28 °C [96]. Furthermore, Abdipour et al. [97] also demonstrated that the application of both UV lights significantly prevent TP content changes, UV-C irradiated sweet cherry fruit showed a higher retention level of TP content than that observed in UV-B irradiated fruit. Conversely, in “Michele Palieri” and “Alphonse Lallée” table grape TP content of uncoated fruit were significantly higher than those treated with CS [98] or CS plus UV-C [99].

However, Chang et al. [100] reported that CS coating combined with hot air, at 37 °C for six hours, had similar protective effects on the increase of TP content of plum fruit compared with uncoated fruit during storage.

CS coating enriched with essential oils such as clove and neem retained the content of TP in lemon and guava fruits during storage [54,101] but several studies have demonstrated that the profile of phenolic compounds changed in CS-EOs coated guava and mango fruits [77,102]. A slower decrease of several phenolic acids and flavonoids such caftaric acid, chlorogenic acid, caffeic acid, narygenin, quercitin 3-glucosyde, and trans-resveratrol has been found in guava fruit coated with CS and lemongrass essential oil [77]. Similarly, in mango fruit coated with CS combined with peppermint essential oil has been highlighted a significant changes in seven different phenolic compounds, such as gallic acid, cis-resveratrol, catechin, procyanidin B1, procyanidin B2, caftaric acid, and caffeic acid compared to uncoated fruit during 30 days at 12 °C [102]. Similarly, fig fruit dipped in composite edible coating with CS/Na-alginate changed the profile of phenolic acids such as chlorogenic acid and gallic acid in maturity stage-dependent manner during 15 days at 6 °C [103]. Pomegranate arils coated with clove- and Satureja hortensis essential oil-CSNPs displayed the highest amount of TP content during 54 days of storage at 5 °C [104] and 18 days of storage at 5 °C [86], respectively.

It has been also reported that, the addendum of olive leaves, pomegranate peel, apple peel, stevia extract and cassava starch into CS coating reduced the gradual decline in TP and/or TF in strawberry [47], guava [82], litchi [105], sweet cherry [106], fresh-cut apple [107], and black mulberry [108] during storage. Furthermore, nanostructured CS/propolis coated strawberry fruits exhibited the highest TP content on each day of cold storage [109]. Similarly, CaCl_2_ (3%) and nano-CS (NCS) coating on strawberry fruit delayed the decline of TP content throughout the storage at 4 °C up to 15 days [49].

Composite cassava starch/CS ECs had no significant effect on phenolic metabolism, without variations on TP content in black mulberry [108]. Instead, whey protein/CS coating enhanced the TP content in stored strawberry at 5 °C and 20 °C for 8 days [110]. Furthermore, CS in combination with aloe vera gel maintained significantly (*p* ≤ 0.05) higher TP content in mango than uncoated fruit during the entire storage period [111]. In mango fruit coated with 1% CS combined with 0.1 ppm spermidine, TP content slightly increased until the end of storage, with higher value than other tested treatments [112].

S-nitrosoglutathione-CS nanoparticles significantly increased the content of TP in apple slices, compared with the control across virtually all examined time points, especially on day 2 (*p* < 0.05) and day 4 (*p* < 0.05) of storage [32].

Anthocyanins are water-soluble pigments belonging to the phenolic compounds group responsible for the bright red color and nutritional quality in several fruits [113].

In CS-coated litchi, sweet cherry, blueberry, strawberry, longan, and pomegranate fruits have been detected a lower reduction of anthocyanin content compared to uncoated ones probably due to their enzymatic degradation owing to anthocyanase, PPO, and peroxidase (POD) throughout the storage period [11,45,49,64,66,67,70,78,83,92,97,104,114].

It has also been observed that in CS-coated plums a lower increase of anthocyanin content during storage ascribable to the suppression of respiratory activity that allows to slow down the anthocyanin’s synthesis with the decrease in the enzymes activity such as PAL and anthocyanidin synthase (ANS) associated with postharvest ripening [56]. Contrarily, in other studies have been observed that CS-based coatings improved the anthocyanin content of strawberry, black mulberry, loquat, fig, sweet cherry, pomegranate, and pomegranate arils [11,45,47,90,94,108,115] suggesting that CS delays fruit senescence and enhances the phytochemicals content during storage. An opposite trend was found by Zam [106] which report that the dense structure of CS-based coating enriched with olive leaves extract reduced the increase of anthocyanins, probably due to changes in the fruit internal atmosphere during storage compared to uncoated fruits.

#### 3.2.2. Enzymatic Antioxidant System

Several studies have investigated the effects of CS-based coatings of the enzymatic components of the anti-oxidative defense system involved in ROS scavenging in different fruit crops during storage [13].

SOD activity increased significantly with higher values in CS coated sweet cherries compared to uncoated ones over 14 days of cold storage. Furthermore, two-fold increase in SOD activity occurred in CS coated fruit during shelf life as compared to cold storage, enhancing the ROS scavenging potential in “Ferrovia”, Lapins”, and “Della Recca” sweet cherries [11]. SOD activity also followed the same trend in CS coated “Sagrantino” grape during partial dehydration [53]. Similarly, in CS/nano-silica coated loquat, SOD activity increased and remained higher than that in uncoated fruit, especially from 20 days to 40 days of storage [37]. An increase in SOD activity were displayed in grape treated with CS coating after 12 days of inoculation with Botritis cinerea with higher value in “Shine Muscat” compared to “Kyoho” grape [38]. Likewise, in CS coated avocado was registered a high SOD activity in artificially or naturally inoculated fruit with either C. gloeosporioides or Lasiodiplodia theobromae after 5 days of shelf life [59]. Otherwise, sweet cherries treated with nitric oxide-releasing CS nanoparticles showed an increase in SOD activity during 25 days of storage at 4 °C, but uncoated fruit displayed higher values compared to treated fruit [39].

In cherimoya fruit, the combined citric acid and CS coating treatment induced the activity of SOD that reached a peak after 2 days and then decreased steadily during the following ten days of storage [41]. Similar trend of SOD activity was reported in salicylic acid (2 mM) and CS (2% *w*/*v*) coated pistachio fruit. SOD activity increased reaching a peak on day 7 and then gradually decreased to day 28 with higher value in coated fruit [95]. These findings were confirmed in guava and maiiayou pummelo treated with CS coating (1.5% *w*/*v*) and stored for 12 days at room temperature and for 150 days at 20 °C, respectively. SOD activity increased gradually up to third day in guava fruit and 105 days in majiayou pummelo, respectively and after decreased as the storage period progressed [40,63]. Furthermore, in CS coated majiayou pummelo, the relative expression of *MnSOD* gene was upregulated from 90 to 150 days of storage period compared with uncoated fruit [40].

In strawberry treated by CS coatings with different MW, SOD activity firstly increased and maintained a stable value, and then gradually decreased with time with higher value in coated fruit. No statically difference were found among CS coatings with different MWs [48]. Fresh-cut apple slices treated with S-nitrosoglutathione-CS nanoparticles showed a rapid decrease in SOD activity during the first day of storage but this value is 79.47% higher than that of uncoated fruit, afterwards, SOD activity rose marginally until to 4 days [32].

CS-based coating in several loquat cultivars and selections did not improve the SOD activity throughout cold storage. SOD activity is influenced by the loquat genotypes and its highest value was found in “Golden Nugget” and “CREAFRC-S38” among all analyzed loquat cultivars and selections, respectively [52].

All analyzed studies demonstrated that CS-based coating treatments in several fruit crops, enhanced SOD activity responsible to scavenge of excessive reactive oxygen species inhibiting free radical accumulation by forming H_2_O_2_ [37,63].

Different trend in CAT and APX activities were found in three strawberry cultivars coated with 1% and 2% CS and stored at 2 °C for nine days. CAT activity decreased during cold storage, but this decrease was higher in uncoated fruit than in coated fruits. CS treatment suppressed this reduction in a dose-dependent manner. Contrarily, APX and GPX activities increased and CS treatment reduced the increase in a dose-dependent manner [45]. Likewise, CS coated sweet cherry fruit displayed an increase of CAT and APX activities during 14 days of storage at 2 °C plus 3 days of shelf-life at 24 °C, with higher valued in coated fruit [57]. Otherwise, in several loquat cultivars and selections treated with CS-based coating (1% *w*/*v*) CAT and APX activity decreased throughout the cold storage with higher values in coated fruit compared to uncoated ones [52]. GPX activity registered lower values in Ch-coated sweet cherry and loquat fruits [52,58]

MWs of chitosan influence in different manner APX activity in nectarine fruit. In high MW-CS and low MW-CS coated fruit, APX was higher 3.60 and 1.57 times and 1.76 and 1.53 times compare to control, at 4 and 8 days of storage, respectively [55]. CS coating with high MW in mango fruit increased the CAT and APX activities up to 4 days of storage reaching the highest values, and then delayed the reduction until the end of storage [8]. Low MW CS elicits plant defensive responses by increasing redox enzyme such as GPX in coated pear fruits [116]. Similar results shown that CS/nano-silica coated loquat enhanced CAT activities reaching a peak value on the fifth and tenth day of cold storage, afterwards, this activity commendably maintained during the remainder storage [37]. Combined citric acid and CS coating treatment in cherimoya fruit showed the same trend in CAT activity that reached the highest value at two days, and then dropped and remained relatively steady for the remainder of the storage period while this postharvest treatment induced the activity of GPX [41]. In pomegranate fruit CS treatment combined with malic and ossalic acid induced an increase in CAT activity up to 90 days of cold storage at 2 °C.

The highest CAT activity was obtained in fruit treated with CS plus ossalic acid (5 mM) with 1.37-fold higher than control after 120 days of storage [42]. Furthermore, in pomegranate arils and pistachio fruit treated with CS-based coatings, CAT activity increased significantly during the first 5 and 7 days of storage, respectively with the highest value in CS treatment, but thereafter its activity declined with higher value in coated fruits [74,95]. Application of CS-SA treatments regulated the balance between ROS generating enzymes, improving the GPX activity in pistachio fruit [95]. Contrarily, CS coating combined with AA significantly suppressed GPX activity in pomegranate arils during the whole storage time compared to other tested treatments [74].

CAT, APX, and GPX activities increased after 12 days of inoculation with B. cinerea in “Shine Muscat” and “Kyoho” grape treated with CS coating, with higher values in “Shine Muscat” [38]. Likewise, a high CAT activity was registered in CS coated avocado fruit artificially or naturally inoculated with either C. gloeosporioides or L. theobromae after 5 days at 18 °C [59].

CAT activity was significantly higher than that in the control fruits for the first three months of storage in CS coated majiayou pummelo fruit with an upregulation and downregulation of CAT encoding gene at 45 and 120 days of storage, respectively. APX activity were significantly higher in CS-coated fruits compared to uncoated ones after 45 days of storage [40].

Contrarily, in CS-based coated fig fruit enriched with AA, CAT activity maintained a stable trend toward storage in coated fruits, while APX and GPX activities significantly increased during cold storage with higher and lower values in CS-coated fruits compared to the uncoated ones, respectively [75].

It has been reported that pre-treatment with S-nitrosoglutathione-CS nanoparticles on fresh-cut apple slices, kept the CAT activity at a higher level compared to other tested treatments, while inhibited the decline of APX activity throughout the whole storage time [32] while nitric oxide-releasing CS nanoparticles improved CAT and GPX activities in sweet cherry treated fruit after 15 days of storage, and slowed down the decrease of APX activity throughout of storage [39]. Furthermore, GSNO-CS NPs treatment maintained higher GPX activity in apple slices avoiding its decrease on the early of storage [32].

CS-based nanoparticles coatings in apple and sweet cherry also induced an increase of enzymes activity involved in AA-GS cycle [32,39]. Fresh-cut apples treated with GSNO-CS NPs displayed higher MDHAR and DHAR activities at the end of storage, allowing a rapid regeneration of AA used as substrate for APX to scavenge H_2_O_2_. This suggests that CS-based treatment regulates both indirect and direct antioxidant systems in fresh-cut apple slices during cold storage [32]. It also been reported that nitric oxide-releasing CS nanoparticles induced an increase in MDHAR and DHAR activity in sweet cherry fruit throughout 25 days of storage with the highest values on day 20. These enzymes responsible to reduction of AA have important roles to maintain high AA pools in a reduced state need to protect against the deleterious effects of ROS [32]. Furthermore, GR activity decreased during storage with higher valued in CS-coated fruit [39]. It has been also reported that CS coating with high MW induced an increase of GR activity in nectarine compared to CS with low MW and uncoated sample [54].

GPX activity showed different trends depending on tested CS-based coatings, storage conditions and fruit crops. Several studies demonstrated that GPX gradually increased in several fruits such as mango, blueberry, and guava during storage [50,73,77,114]. It has also been reported that 3% CS-based coating induced GPX activity in guava compared to other tested CS concentrations, counteracting the injury due to excessive levels of ROS. In agreement with this study, CS coating plus silicon dioxide nanoparticles and nisin applied on fresh blueberry induced GPX activity that reaches the highest value (37.75 U min^−1^g^−1^) after 8 days of storage [114]. Likewise, CS coated fresh cut lemon slices stored at different temperatures showed an increase in GPX activity at high temperature, suggesting that low temperature (0 °C) had an excellent inhibitory effect on the activity of GPX [54]. Contrarily, in longan fruit, the application of different UV-C and CS-based coating combinations had no significant effect on GPX activity on both pericarp and flesh during seven days of storage [96]. In guava fruit, composite coating containing CS/Nano-TiO_2_ and CS and lemongrass essential oil inhibited GPX activity during storage with lower and higher values in coated fruit at 10 and 15 days of storage, respectively [51,77].

CS-based coatings enhanced the antioxidant systems in fruit crops during storage, inducing non-enzymatic antioxidants biosynthesis, promoting the bidirectional recycling of antioxidants such as ASA-GSH cycling and enhancing antioxidant enzymes activity.

## 4. Enzymatic Browning

Fruit browning is mainly due an enzymatic oxidation of phenolics to quinones mediated by polyphenol oxidase (PPO) that reduce the consumer’s acceptability causing over 50% of loss in fruits [117]. Several studies have demonstrated that CS-based coatings has an inhibitory effect on PPO activity in different fruit crops such as strawberry [45], sweet cherry [57], cherimoya [41], loquat [37,51], longan [96], guava [58,77], grape [53,84], fig [75], apple [32,107], mango [50,102], pistachio [95], lemon [54], and pineapple [89]. These findings could be due to low O_2_ availability, inside the fruit, necessary for initiate the browning reactions. Furthermore, the maintenance of membrane integrity due to the balance between ROS- production and scavenging systems in CS-coated fruit suggests a preservation of cell compartmentalization and separation of PPO from their phenolic substrates [52,57].

## 5. Conclusions and Future Trends

CS-based coatings as mono- and bilayer represent a valid tool to prolong the postharvest life of several whole and fresh-cut fruits. This alternative strategy delays the changes typically associated with ROS network improving non-enzymatic and enzymatic antioxidant systems. Several studies have shown a maintaining the balance of intracellular oxidation metabolism in CS-coated fruit due to capable of efficiently clearing cytotoxic compounds via the enzymatic antioxidant such as CAT, APX, GPX, MDAR, DHAR, GR, and non-enzymatic antioxidants such as GSH, AA, phenols, anthocyanins, and flavonoids. Furthermore, the maintenance of membrane integrity in CS-coated fruit, confirmed by lower LOX activity and MDA content, suggests a preservation of cell compartmentalization and separation of PPO enzyme from their phenolic substrates with low enzymatic browning.

In addition, CS-based coatings tested on a laboratory scale in several fruit crops should be needed to be investigated further to large-scale tests with potential commercial applications.

## Data Availability

This study did not report any data.

## Supplementary Materials

The following are available online at https://www.mdpi.com/1422-0067/22/5/2633/s1, Table S1: Studies of the last five years related to the effect of chitosan (CS)-based coatings on antioxidant systems and enzymatic browning in several fruit crops during several storage conditions.
