# Supplementary material for: Multifunctional Role of Chitosan Edible Coatings on Antioxidant Systems in Fruit Crops: A Review
Source: Int J Mol Sci. 2021 Mar 5;22(5):2633. doi: 10.3390/ijms22052633 (PMC7961546; doi:10.3390/ijms22052633)
Supplement: Supplementary file 1 [file ijms-22-02633-s001.pdf]

Table S1. Studies of the last five years related to the effect of chitosan (CS)-based coatings on antioxidant systems and enzymatic browning in several fruit crops during several storage conditions.

	**Fruits**	**CS-based coating**	**Coating method**	**Storage conditions**	**Effect on antioxidant systems and enzymatic browning**	**Reference**
1.	Kiwifruit cv. 'Hayward' (fresh-cut)	CS 1% (w/v) (formulated with acetic (AC; 1% v/v) or citric acid (C; 2 % w/v))	Dip coating	4 °C and 75 % RH for 12 days	↓ Ascorbic acid content (CS + AC)<br>→ Ascorbic acid content (CS + C)	[81]
2.	Mango cv. 'Kent' (whole)	CS (1 and 1.5% w/v) with lactoperoxidase and iodine	Dip coating	18 °C and 60 % RH for 8 days	↑ Ascorbic acid content	[76]
3.	Strawberry cvs. 'Candonga', 'Ionica' and 'Sabrina' (whole)	CS 1% and 2 (w/v)	Dip coating	2 °C and 95 % RH for 9 days	↑ Ascorbic acid content, total phenolics, total flavonoids, total anthocyanins, antioxidant activity<br>↑ CAT activity<br>↓ APX and LOX activity<br>↓ PPO and GPX activity<br>↓ MDA content	[45]
4.	Litchi cv. 'Purbi' (whole)	CS (2% w/v) with salicylic acid (0.5 mM and 1.0 mM)	Dip coating	4 °C for 6 days	↑ Ascorbic acid, total phenolics, total flavonoids, anthocyanins, antioxidant activity	[78]
5.	Mango cv. 'Tommy Atkins' (fresh-cut)	CS (0.2 % w/v) and Na-alginate (0.2 % w/v) (five nanolayers)	Dip coating	8 °C and 93 % RH for 14 days	→ Ascorbic acid content<br>↓ Malondialdehyde content	[50]
6.	Loquat Selection (CREAFRC-S18; CREAFRC-S35 and CREAFRC-S36) cvs (Golden Nugget, Algerie and Nespolone rosso di Trabia) (whole)	CS 1 % (w/v)	Dip coating	7 °C and 95 % RH for 21 days	↑ Ascorbic acid content, total phenolics, total flavonoids, antioxidant activity<br>↓ Carotenoids content,	[71]

7.	Sweet cherry cvs. 'Ferrovia', 'Lapins' and 'Della Recca' (whole)	CS 0.5 % (w/v)	Dip coating	2 °C and 95 % RH for 14 days plus 24 °C for 3 days	↑ SOD, APX, CAT activity<br>↓ PPO, LOX, POD activity<br>↓ MDA activity	[57]
8.	Sweet cherry cvs. 'Ferrovia', 'Lapins' and 'Della Recca' (whole)	CS 0.5 % (w/v)	Dip coating	2 °C and 95 % RH for 14 days plus 24 °C for 3 days	↑ Ascorbic acid, total phenolics, total flavonoids, anthocianins, antioxidant activity	[11]
9.	Mango cv 'Nam Dok Mai' (whole)	CS (1 % w/v) with high, medium and low molecular weight	Dip coating	25 °C for 16 days	↓ H_2O_2 content<br>→ Ascorbic acid, total phenolics, total flavonoids, antioxidant activity<br>→ CAT and APX activities	[8]
10.	Red Kiwifruit (whole)	CS (1 % w/v)	Dip coating	20 °C for 26 days	↑ Ascorbic acid, total phenolics	[69]
11.	Apple cv. 'Anna' Strawberry cv. 'Festival' (whole)	CS (2 % w/v) with olive leaf extracts (1-2 % w/v) and olive pomace extracts (1-2 % w/v)	Spray coating	4 °C for 35 and 16 days for apple and strawberry, respectively	↓ MDA content	[46]
12.	Strawberry cv. 'Festival' (whole)	CS (2 % w/v) with olive leaf extracts (1-2 % w/v) and olive pomace extracts (1-2 % w/v)	Spray coating	4 °C for 16 days	↑ Ascorbic acid, total phenolics total flavonoids, anthocianins, antioxidant activity<br>↓ MDA content	[47]
13.	Cherimoya (whole)	CS 1.0 % w/v with 20 mM citric acid	Dip coating	15 °C for 10 days	↑ Ascorbic acid content<br>↑ CAT, SOD, POD activity<br>↓ PPO activity, $O_2^{\bullet -}$ and MDA content	[41]
14.	Apple cv. 'Fuji' (fresh-cut)	CS 0.2 % (w/v) with ascorbic acid 0.2 % (w/v)	Dip coating	Room temperature for 10 hours	↑ Ascorbic acid content	[79]

		or calcium chloride 0.2 % (w/v)				
15.	Pomegranate cv 'Mallas Saveh' (whole)	CS 0.5 % (w/v), salicylic acid 2 mM, and salicyloyl CS 0.57 %	Dip coating	2 °C for 5 months plus 3 days at 20 °C	↑ Total hydrophilic and lipophilic antioxidant activity, total phenols and total anthocyanins	[94]
16.	Loquat cv 'Baiyu' (whole)	CS 0.75 %, w/v nano-silica 30%, w/v	Dip coating	5 °C for 40 days	↓ $O_2^{\bullet-}$, H_2O_2 and MDA content<br>↓ PPO and LOX activity<br>↑ CAT, SOD activity	[37]
17.	Kiwifruit cvs. 'Anykista', 'Sentiabrskaya' and 'VIR2' (whole)	CS 1 % (w/v) with high, medium and low molecular weight	Dip coating	Room temperature for 14 days	→ Ascorbic acid, total phenols	[91]
18.	Mango cv 'Nam Dok Mai' (whole)	CS 1 % (w/v) with 0.1 ppm spermidine	Dip coating	25 °C for 9 days	→ H_2O_2 content, total phenolics	[112]
19.	Plum (whole)	CS 2 % (w/v)	Dip coating	1 °C and 90 % RH for 35 days	↓ MDA content and anthocyanins<br>↑ Total phenols and ascorbic acid	[56]
20.	Guava cv. 'Pant Prabhat' (whole)	CS 0.5, 1 and 1.5 % (w/v)	Dip coating	27-29°C and 70-75% RH for 12 days	↑ Total flavonoids<br>↑ Total phenols<br>↑ SOD activity<br>↓ Antioxidant activity	[63]
21.	Longan cv. 'Diamond' (whole)	CS 1.29 % (w/v) followed by UV irradiation	Dip coating	28 °C for 7 days	↑ Total phenols<br>↓ PPO, POD activity	[96]
22.	Pomegranate cv. 'Herskawitz' and 'Wonderful' (arils)	CS 0.25 %, 0.75 % and 1.5 % (w/v)	Dip coating	4 °C and 95 % RH for 14 days	↑ Ascorbic acid, antioxidant activity, total phenols and total anthocyanins	[64]
23.	Table grape cv. 'Yongyou 1' (whole)	CS 1 % (w/v) and CS-g-salicylic acid conjugate 1.03 % (w/v)	Spray coating	0 °C and 90-95% RH for 42 days	↑ Total phenols	[93]

24.	Pear cv. 'Huangguan' (whole)	CS 5% (w/v)	Dip coating	25 °C for 21 days	↑ PPO and POD activity<br>→ Ascorbic acid	[117]
25.	Pineapple (fresh-cut fruit)	CS and pullulan, CS and mucilage (obtained by linseed, nopal cactus and aloe)	Dip coating	4 °C for 18 days	↑ Ascorbic acid content	[86]
26.	Kumquat (whole)	CS 0.4% (w/v) with savory (1.25, 2.5 ppm) and tarragon oil (1.25, 2.5 ppm)	Dip coating	7 °C for 30 days	↑ Ascorbic acid	[80]
27.	Guava cv. 'Banati' (whole)	CS 1% (w/v) with poly-vinyl-pyrrolidine 1 % (w/v) and salicylic acid (1 and 2 mM)	Dip coating	27 °C and 48% RH for 15 days	↑ Ascorbic acid, total phenolic, antioxidant capacity<br>↓ PPO and LOX activity	[58]
28.	Pomegranate cv. 'Hicnazar' (whole)	CS 1% (w/v)	Dip coating	6 °C and 90 % RH for 6 months with or without modified athmosphere packaging	↑ Ascorbic acid, total monomeric anthocyanin, total phenolic, antioxidant capacity (Ferric reducing antioxidant power)<br>→ Antioxidant capacity (Trolox equivalent antioxidant capacity)	[70]
29.	Litchi cv. 'Wuye' (whole)	CS 2% (w/v)	Dip coating	25 °C and 85 % RH for 6 days	↑ Ascorbic acid, total anthocyanin, total phenolic, total flavonoids	[66]
30.	Guava cv. 'Allahabad' (whole)	CS solution 1 % (w/v) with and without pomegranates peel extract 1%	Dip coating	10 °C and 90-95 % RH for 20 days	↑ Ascorbic acid, total flavonoids, total phenolic, antioxidant capacity (DPPH and FRAP)	[82]
31.	Guava cv. 'Paluma' (whole)	CS 1, 2 and 3 % (w/v)	Dip coating	25 °C and 85 % RH for 96 hours	↓ Ascorbic acid<br>↑ POD activity	[73]
32.	Table grape cv. 'Kyoho' (whole)	CS 1.5 % (w/v) with lauroyl arginate ethyl 0.075 % (w/v) and montmorillonite 0.1 % (w/v)	Dip coating	4 °C and 95 % RH for 20 days	↑ Ascorbic acid<br>↓ PPO activity	[84]

33.	Tangerine (whole)	CS (C) 1.5 % (w/v) with montmorillonite (MMT) 0.5, 1 and 2 % (w/w)	Dip coating	10 °C and 70 % RH for 11 days	↑ Ascorbic acid (C+MMT-2 %)	[85]
34.	Loquat Selection (CREAFRC-S18; CREAFRC-S35 and CREAFRC-S36) cvs (Golden Nugget, Algerie and Nespolone rosso di Trabia) (whole)	CS 1 % (w/v)	Dip coating	7 °C and 95 % RH for 21 days	↑ CAT and APX activity<br>↓ SOD activity<br>↓ LOX, PPO and POD activity<br>↓ MDA content	[52]
35.	Grape cv 'Sagrantino' (whole)	CS 1 and 2 % (w/v)	Dip coating	20 ° C and 60 % RH	↑ Total phenolics<br>↑ SOD and APX activity<br>↓ LOX and PPO activity<br>↓ MDA content	[53]
36.	Fig cv 'Troiano' (whole)	CS 1 % (w/v) with ascorbic acid 1 % (w/v)	Dip coating	4 °C and 95 % RH for 9 days	↑ Ascorbic acid content, total phenolics, total flavonoids, antioxidant activity<br>↑ CAT and APX activity<br>↓ PPO and GPX activity	[75]
37.	Pomegranate cv. 'Rabbab-e-Neyriz' (whole)	CS 2 % (w/v) with and without malic (50 and 100 mM) and ossalic (5 and 10 mM) acid	Dip coating	2 °C at 80 %–90 % RH 120 days	↑ Total phenols, ascorbic acid and antioxidant activity<br>↓ H_2O_2 and MDA content<br>↑ CAT activity<br>↓ PPO and POD activity	[42]
38.	Plum cv. 'Sanhua' (whole)	CS (1.0 w/v) and hot air (37 °C for 6 hours)	Dip coating	5 °C for 40 days	↑ Total phenols and flavonoids (until to 24 days)<br>→ Antioxidant activity	[100]
39.	Nectarine (Whole)	CS (1.0 w/v) with 30 and 120 kDa MW	Dip coating	28 °C at 80-90 % RH 8 days	↓ H_2O_2 content (CS-120)<br>↓ MDA content<br>↑ APX, GR activity<br>↑ GSH, ASA content	[55]

					↑ Total phenol, flavonoids (CS-120)<br>↑ Antioxidant activity	
40.	Peach (whole)	CS (1.0 w/v) chlorogenic acid (1:1, 1:0.5, or 1:0.1 molar ratios) conjugate	Dip coating	20 ° C for 8 days	↑ Ascorbic acid<br>↓ ROS content	[43]
41.	Pineapple cv. 'Yellow Mauritius' (fresh-cut)	CS (1.0 w/v) grafted with procyanidin (1:0.8)	Dip coating	4°C for 14 days	↑ Ascorbic acid, total phenols, antioxidant activity<br>↓ PPO and POD activity	[89]
42.	Apple cv. 'Amasya' (Fresh-cut)	CS (0.25 w/v) with and without 2.5 (w/v) Stevia extract	Dip coating	1 °C in Passive MAP (the beginning gas composition: 21% O_2, 0.03% CO_2 and other gases) for 3 days	↓ PPO activity<br>↑ Total phenols, antioxidant activity	[107]
43.	Sweet cherry cv. 'Hongdend' (whole)	CS (0.5 w/v) with 60 mM S-nitrosoglutathione (nanoparticles)	Dip coating	0 °C for 25 days	↓ ROS content<br>↑ SOD, POD, CAT, GR, DHAR, MDAR, APX activity<br>↑ ASA, GSH content<br>↓ DHA, GSSG content	[39]
44.	Avocado cv 'Hass' (whole)	CS 1 and 1.5 w/v	Dip coating	7.5 °C for 28 days plus 5 or 7 days at 18 °C	↑ SOD, CAT activity<br>↓ LOX activity<br>↑ Epicatechin content	[59]
45.	Apple cv. 'Granny Smith' (fresh-cut)	CS 1 % w/v and ascorbic acid 1 and 5 % w/v<br>CS 2 % w/v and ascorbic acid 2 % w/v	Dip coating	5 °C for 18 days	↑ Catechin, epicatechin and chlorogenic acid content CS 1 % + AA 5 %)	[92]

46.	Fig (whole)	CS 1 % (w/v) and Na-alginate 2 % (w/v) (layer-b-layer)	Dip coating	6 °C at 95 % RH for 15 days	→ Total phenolic, antioxidant activity	[103]
47.	Table grape cv. 'Alphonse Lavallée' (whole)	CS 0.5, 1 and 2 % w/v	Dip coating	1 °C at 90 % RH for 28 days	↓ Total phenols<br>↑ Antioxidant activity (CS-2 %)	[98]
48.	Fig cv. 'Siah' (whole)	CS 0.5 % w/v with and without Thymol 200 ppm	Dip coating	6 °C at 80 % RH for 20 days	↑ Anthocyanin content	[115]
49.	Mango	CS 0.2 % (w/v) and Na-alginate 0.2 % (w/v) with cinnamon essential oil (three, five and seven layers)	Dip coating	25 °C at 50 % RH for 14 days	↑ Ascorbic acid content	[19]
50.	Sweet cherry cv. 'Burlat' and 'Bigarreau' (whole)	Na-alginate 3 % w/v and CS 1 % w/v with and without olive leaves extract 1 % w/v	Dip coating	25 ° C at 65 % RH for 20 days	↑ Ascorbic acid content, total phenols, antioxidant activity<br>↓ Anthocyanin content	[106]
51.	Papaya cv 'Lingnanzhong' (whole)	CS 0.5 w/v and pullulan 0.5 5 w/v (two, four and six layers)	Dip coating	25 ° C at 50 % RH for 14 days	↑ Ascorbic acid content	[20]
52.	Sweet cherry cv. 'Takdaneh Mashhad' (whole)	CS 1% (w/v) with UV-B and/or UV-C radiation	Dip coating	5 °C with 90 % RH for 28 days.	↑ Ascorbic acid content, total phenolics, anthocyanins, antioxidant activity	[97]
53.	Mango cv. 'Tommy Atkins' (whole)	CS 0.5 % (w/v) with peppermint (1.25 and 0.6 mL L−1)	Dip coating	12 °C with 85% RH for 30 days	↑ Total phenolics<br>↓ POD and PPO activity	[102]
54.	Blueberry (whole)	CS 1% (w/v) plus silicon dioxide nanoparticles and nisin (1%)	Dip coating	28 °C with 75% RH for 8 days	↑ Ascorbic acid content, anthocyanins<br>↑ POD and PPO activity	[114]
55.	Pistachio	CS 2% (w/v) with and without salicylic acid (2 mmol L^{-1}),	Dip coating	4 °C with 90–95% RH for 28 days	↑ Total phenolics<br>↑ SOD, CAT and POD activity<br>↓ PPO activity	[95]

56.	Majiayou pummelo (whole)	CS 1.5% (w/v)		20 °C for 150 days	↑ Ascorbic acid and glutathione coO2<br>↓ $O_2^{-}\cdot$, H_2O_2 and MDA content<br>↑ SOD and APX activity	[40]
57.	Mango cv. 'White Chaunsa' (whole)	CS 1 % (w/v) with and without aloe vera gel	Dip coating	12 °C with 80–85%for 28 days plus 25 °C for 5 days	↑ Ascorbic acid content, total phenolics, antioxidant activity	[111]
58.	Mango (whole)	CS 1 % (w/v) /Nano-TiO_2 (0.01 g and 0.03 g)	Dip coating	13 °C	↑ Total phenolics, total flavonoids<br>↓ MDA content<br>↓ PPO and POD activity	[51]
59.	Lemon cv. 'Eureka' (fresh-cut)	CS 1.23 % (w/v) with clove oil 0.10 %	Dip coating	0 °C, 4 °C, 7 °C and 10 °C for 10 days	↑ Total phenolics, total flavonoids<br>↓ MDA content<br>↓ PPO and POD activity	[54]
60.	Mandarin 'Kinnow' (whole)	CS 0.05, 0.1, 0.15 % (w/v)	Dip coating	5-7 °C at 90-95 % RH per 75 days	↑ Ascorbic acid, total carotenoid	[68]
61.	Lemon (whole)	CS 1.5 % (w/v) and CS 1.5 % (w/v) - carboxymethyl cellulose 1.5 % (w/v)	Dip coating (layer-by-layer)	0 °C for 20 days	↑ Ascorbic acid	[24]
62.	Guava cv. 'Paluma' (whole)	CS 0.5 % (w/v) with or without lemongrass essential oil 0.6 ppm	Dip coating	12 °C for 15 days	↑ Caftaric acid, Chlorogenic acid, Caffeic acid<br>↑ Narygenin, Quercetin 3-glucosyde, Trans-resveratrol<br>↑ POD activity<br>↓ PPO activity	[77]
63.	Pomegranate cv. 'Malase Saveh' (arils)	CS 0.3 % (w/v) nanoparticles with and without clove essential oil 0.15 % (w/v)	Dip coating	5 °C at 90 % RH for 60 days	↑ Total phenols, anthocyanins, and antioxidant activity	[104]

64.	Strawbeery (whole)	CS 1.2 % (w/w) with different molecular weight (5, 19 and 61 kDa)	Electrostatic spraying coating	4 °C at 85 % RH for 15 days	↑ Total flavonoids<br>↓ MDA content<br>↑ SOD activity	[48]
65.	Sweet orange cv. 'Baladì' (whole)	CS 0.25, 0.5 and 1 % (v/v)	Dip coating	4° C at 85-90 % RH for 8 weeks	↑ Ascorbic acid	[72]
66.	Litchi cv. 'Deshi' (whole)	CS 2 % and pullulan 2 % (50:50) with pomegranate peel extract	Dip coating	23 ºC at 40-45% RH for 18 days<br>4 ºC at 90-95% RH for 18 days	↑ Total phenolic, total flavonoids, antioxidant activity	[105]
67.	Longan cv. 'Fuyan' (whole)	CS 0.4, 0.2, 0.13, 0.1 % (v/v)	Dip coating	25 °C at 85% RH for 6 days.	↑ Carotenoids and total phenols<br>↑ Anthocyanins (0.2 and 0.13 %)<br>↑ Total flavonoids and ascorbic acid (0.2 %)	[67]
68.	Strawberry cv. 'Camarosa' (whole)	CS 1 % (w/v), CS with CS nanoparticles (CSNPs) (33 %), CS with CS nanoparticles with propolis (P) (10-30 %)	Dip coating	4 °C for 8 days	↑ Total phenols and antioxidant activity<br>↑ Total flavonoids (CS + CSNPs + P 20%)	[109]
69.	Strawberry (whole)	CS (1 %) with and without whey protein (1%)	Dip coating	5 °C and 20 °C, at 75 % RH for 8 days	↑ Total phenols, ascorbic acid and antioxidant activity	[110]
70.	Strawberry (whole)	CS nanoparticles (NCTS) (0.2 %) with $CaCl_2$ (0-4 %)	Dip coating	4 °C for 15 days	↑ Ascorbic acid, total phenols, anthocyanins, antioxidant activity (NCTS+ $CaCl_2$ 3 % and (NCTS+ $CaCl_2$ 4 %)<br>↓ MDA content (NCTS+ $CaCl_2$ 3 % and (NCTS+ $CaCl_2$ 4 %)	[49]
71.	Black mulberry (whole)	CS 1 % (w/v) with cassava starch 1 % (w/v)	Dip coating	5 °C for 16 days	↑ Total phenols, anthocyanins<br>→ Antioxidant activity	[108]
72.	Strawberry cv. 'Hongyan' (whole)	CS 1 % (v/v) with apple peel polyphenols 0.25, 0.5, 0.75 and 1 % (w/v)	Dip coating	20 °C at 35-40% RH for 6 days	↑ Total phenols, total flavonoids, anthocyanins, ascorbic acid, antioxidant activity	[83]

73.	Sweet cherry cv. 0900-Ziraat (whole)	CS (CH-1) and (CH-2) extracted by shrim waste, commercial CS (CS-1, deacetylation degree: 81.22%, molecular weight: 273 kDa; CS-2, deacetylation degree: 75.12%, molecular weight: 407 kDa)	Dip coating	4 °C and for 25 days<br>20 ° C for 15 days	↑ Ascorbic acid and antioxidant activity (C1; 4 °C)<br>↑ Anthocyanins, total phenols (CS-2; 4 ° C)<br>↑ Total phenols (CS-1; 20 ° C)<br>↑ Anthocyanins (CS-2; 20 ° C)<br>↑ Ascorbic acid (CS-2; 4 °C)	[90]
74.	Apple cv-Fuji (fresh-cut)	CS 0.5 % (w/v) and S-nitrosoglutathione-CS nanoparticles (1 mM)	Dip coating	4 °C for 4 days	↑ Ascorbic acid, total phenols<br>↓ •O_2^- content and PPO activity<br>↓ H_2O_2 content and DHA<br>↑ CAT, SOD, APX, POD, DHAR, MDHAR activity	[32]
75.	Pomegranate cv. ‘Jahrom’ (arils)	CS (CS) (0.5 and 1% w/v), ascorbic acid (1 and 2% w/v), and citric acid (1 and 2% w/v)	Dip coating	5–7 °C at 95% RH for 15 days	→ Ascorbic acid, total phenol, anthocyanins (CS)<br>↓ Antioxidant activity<br>↓ POD activity (CS-1 %)<br>↓ PPO activity (CS-0.5 and 1 %)<br>↑ CAT activity (CS-0.5 and 1 %)	[74]
76.	Guava cv. ‘Paluma’ (whole)	CS (CS) (0.5 and 1% w/v) with and without neem oil (NO) (0.5-1 % v/v)	Dip coating	24 °C for 10 days plus 20 days at 10 °C with 85 % RH	→ Ascorbic acid (CS 1 %, CS 1% + NO 0.5 %, CS 1 % + NO 1 %) at 24 °C<br>→ Total Phenols (CS 0.5 %, CS 1 %, CS 0.5 5+ NO 1 %) at 24 ° C<br>↑ Antioxidant activity (CS 1 % + NO 1 %) at 24 °C<br>↑ POD activity (CS 0.5 %, CS 1 %, CS 0.5% + NO 1 %)<br>→ Ascorbic acid (at 10 °C)<br>→ Total Phenols (CS 1 % + NO 1 %; CS 1 % + NO 0.5 %) at 10 °C	[101]

					↑ Antioxidant activity (CS 1%, CS 0.5 % CS 0.5% + NO 1 %, CS 0.5% NO 0.5 %) at 10 °C<br>→ POD activity (at 10 °C)	
77.	Pomegranate cv. 'Rabab' (arils)	*Satureja hortensis* essential oil-loaded CS (0.5 w/v) nanoparticles (1:0.5)	Dip coating	5 °C at 90 % RH for 18 days	↑ Ascorbic acid, total phenols, anthocyanins, antioxidant activity	[87]
78.	Table grape cv. 'Michele Palieri' (whole)	CS (1 w/v) plus UV-C	Dip coating	1°C at 90% RH for 21 days	↓ Total phenols<br>↑ Antioxidant activity	[99]
79.	Grape cvs. 'Kyoho' and 'Shine Muscat' (whole)	CS (1.5 w/v)	Dip coating	22 °C at 95% RH for 12 days	↑ SOD, CAT, APX, POD activity<br>↓ MDA content	[38]
At the end of storage has been evaluate decrease (↓), increase (↑) or no significant effect (→) on each feature in CS-based coated fruits.						
